# Humoral and Cellular Immune Responses Following Bivalent SARS-CoV-2 mRNA Vaccines in Patients Undergoing Hemodialysis: A Prospective Cohort Study

**DOI:** 10.1016/j.xkme.2026.101480

**Published:** 2026-07-23

**Authors:** Tsai-Chieh Ling, Po-Lin Chen, Jen-Ren Wang, Wen-Chien Ko, Chiao-Hsuan Chao, Chi-Chang Shieh, Jia-Ling Wu, Chien-Yao Sun, Wei-Ren Lin, Chieh-Hsin Huang, Yu-Tzu Chang

**Affiliations:** 1 Department of Internal Medicine, National Cheng Kung University Hospital, College of Medicine, National Cheng Kung University, Tainan, Taiwan; 2 Department of Microbiology and Immunology, College of Medicine, National Cheng Kung University, Tainan, Taiwan; 3 Department of Medicine, College of Medicine, National Cheng Kung University, Tainan, Taiwan; 4 Department of Medical Laboratory Science and Biotechnology, College of Medicine, National Cheng Kung University, Tainan, Taiwan; 5 National Institute of Infectious Diseases and Vaccinology, National Health Research Institutes, Tainan, Taiwan; 6 Department of Optometry, MacKay Medical College, New Taipei City, Taiwan; 7 Department of Pediatric, National Cheng Kung University Hospital, College of Medicine, National Cheng Kung University, Tainan, Taiwan; 8 Institute of Clinical Medicine, College of Medicine, National Cheng Kung University, Tainan, Taiwan; 9 Department of Public Health, College of Medicine, National Cheng Kung University, Tainan, Taiwan; 10 Department of Geriatrics and Gerontology, National Cheng Kung University Hospital, College of Medicine, National Cheng Kung University, Tainan, Taiwan

**Keywords:** Bivalent vaccine, cellular immunity, COVID-19, hemodialysis, humoral immunity

## Abstract

**Rationale & Objective:**

Hemodialysis (HD) patients are susceptible to severe illness and mortality from coronavirus disease (COVID)-19. We investigated immunogenicity following bivalent vaccines containing ancestral and omicron variant (BA.1 or BA.4/BA.5) antigens in HD patients.

**Study Design:**

A prospective observational cohort study.

**Setting & Participants:**

We enrolled adult HD patients and age-matched healthy adults between October 2022 and February 2023. Blood samples were taken at baseline, 1 month (M1), and 3 months (M3) postvaccination.

**Exposures:**

Bivalent mRNA vaccines

**Outcomes:**

Anti-SARS-CoV-2 spike protein receptor-binding domain antibodies, surrogate viral neutralization tests, pseudovirus neutralization tests, and SARS-CoV-2-specific interferon-γ release assay.

**Analytic Approach:**

Continuous variables were assessed using *t* test, Mann–Whitney *U* test, or Kruskal–Wallis test with Dunn’s post hoc test; categorical variables were assessed using chi-square test or Fisher exact test. Holm–Bonferroni correction was applied for multiple comparisons.

**Results:**

Among 106 HD patients, 89.6% and 10.4% received either Spikevax Original/Omicron BA.1 or BA.4/5. Prior SARS-CoV-2 infection was documented in 25.5%, and 93.4% had received four monovalent vaccine doses. Anti-SARS-CoV-2-S antibody levels demonstrated a 4.2-fold increase at M1, subsequently declining by 50% at M3. Surrogate viral neutralization tests showed enhanced neutralization against Omicron BA.1, BA.2, and BA.4/5, with seropositive rates of 75.3% to 96% maintained at M3. Infection-naïve patients exhibited lower baseline antibodies but demonstrated more pronounced booster responses compared with previously infected individuals. Pseudovirus neutralization tests demonstrated 2-to-9.9-fold increases at M1 in neutralizing capacity against emerging Omicron subvariants BF.7, BA.2.75, BQ.1.1, and XBB.1.5. However, only 58.1% of patients demonstrated positive SARS-CoV-2-specific interferon-γ release assay responses at M1, with no correlation to humoral outcomes. Immune responses were comparable between HD patients and healthy controls.

**Limitations:**

Limited sample size, heterogeneous vaccination and infection history.

**Conclusions:**

Bivalent mRNA vaccines significantly enhanced antibody titers and broadened neutralizing capacity against emerging Omicron subvariants in HD patients. However, cellular immunity remained suboptimal despite multiple antigen exposures, highlighting the need for optimized vaccination strategies in this immunocompromised population.

## Plain-Language Summary

Patients receiving hemodialysis are at higher risk for severe coronavirus disease (COVID)-19 outcomes. We studied how well the updated bivalent COVID-19 vaccines (containing both the ancestral virus and Omicron variants) work in 106 hemodialysis patients. We measured antibody levels and T cell responses before vaccination and at 1 and 3 months postvaccination. Results showed that these vaccines effectively increased antibody levels and improved protection against multiple Omicron variants, including newer strains like BF.7 and XBB.1.5. The antibody responses in hemodialysis patients were similar to those in healthy individuals. However, only about 60% of patients developed positive T cell immune responses, suggesting that current vaccination strategies may need further optimization for this vulnerable population.

Patients with kidney disease exhibited elevated risk of severe acute respiratory syndrome coronavirus 2 (SARS-CoV-2) infection, hospitalization, mortality and long coronavirus disease (COVID). Paradoxically, they were less likely to receive advanced therapies like remdesivir or monoclonal antibody therapy.^1^ These findings underscore the critical importance of targeted preventive strategies, with vaccination emerging as a key measure to mitigate infection risks and severe outcomes in this vulnerable group.

As SARS-CoV-2 evolved, especially with the emergence of the Omicron era, new variants exhibit substantial escape from antibody neutralization (acquired either from vaccination or infection), posing a significant threat to effectiveness of existing monovalent vaccines and monoclonal antibody preparation. In response, the Centers for Disease Control and Prevention recommended the bivalent vaccines containing mRNA directed against both the ancestral strain and Omicron subvariants in September 2022. However, the subsequent researches in the general population failed to conclusively demonstrate significantly stronger neutralization against variants compared with monovalent boosters, possibly because of immune imprinting from prior natural infection and monovalent vaccination, which may limit the immune system’s ability to mount novel responses against variant-specific antigens.^2^^,^^3^ Furthermore, by the time bivalent vaccines were developed and approved, the circulating strains had already diverged from the subvariants contained in the vaccines. This led to the postulation that the bivalent boosters might be best reserved for immunocompromised individuals or those with multiple comorbid conditions to prevent severe disease. Recent systemic reviews and meta-analyses showed BA.1 type or BA.4/5 type bivalent mRNA vaccines demonstrated higher effectiveness in preventing symptomatic infection or severe disease compared with monovalent boosters in general population.^4^^,^^5^ However, information on immunogenicity of bivalent boosters in hemodialysis (HD) patients remains scanty, with most studies involving small sample size and short follow-up periods. Vaccine-induced immunity encompasses both humoral responses that block infection and cellular immunity that prevents severe disease, with the latter being particularly important for protection against variants with significant antibody escape such as omicron.^6^ Knowledge gap persists regarding neutralization against omicron subvariants, trajectory of antibodies decay and cellular immunity in this population. We thus conducted a prospective observational study to investigate the magnitude, durability and breadth of humoral and cellular immunity following bivalent boosters in a HD cohort. This study involved a 9-month follow-up period and primarily included patients who had previously received four monovalent vaccinations, with the primary series consisted of two-dose AZD1222.

## Materials and Methods

### Study Design and Study Cohort

This prospective observational cohort study was conducted in National Cheng Kung University Hospital, a tertiary medical center in southern Taiwan. We enrolled maintenance HD patients and monitored their immune responses following SARS-CoV-2 vaccination, beginning with the first dose in June 2021. The immunogenicity following the primary series with AZD1222 and mRNA monovalent boosters have been previously reported.^7^^,^^8^ The current study included individuals age ≥18 years who had been on maintenance HD for more than three consecutive months and received the mRNA-1273 bivalent vaccine (Original and Omicron BA.1 or Original and Omicron BA.4/BA.5) between October 2022 and February 2023. A control group of healthy individuals without kidney failure were also recruited. Blood samples were collected at baseline (before bivalent vaccination) and 1 and 3 months postvaccination (baseline, M1, and M3, respectively) to evaluate immune responses (Fig S1). The follow-up period extended to 9 months post-bivalent vaccination. To detect asymptomatic infections, anti- SARS-CoV-2 nucleocapsid protein (N) antibodies were measured using Elecsys Anti-SARS-CoV-2 assay at baseline and M3. All participants provided written informed consent, and the study protocol was approved by the Institutional Review Board at NCKUH (IRB B-ER-109-024) and all procedures were in accordance with the principles of the 1964 Helsinki Declaration and its later amendments or comparable ethical standards.

### Anti-SARS-CoV-2 Spike Protein (S) Receptor-Binding Domain (RBD) Antibodies

Anti-SARS-CoV-2-S RBD antibodies concentrations were measured at baseline, M1, and M3 using the Elecsys Anti-SARS-CoV-2 S (Roche Diagnostics, Basel, Switzerland), a United States Food and Drug Administration (FDA)-approved electrochemiluminescence immunoassay. It quantifies the levels of total antibodies, including IgG, IgA, and IgM, directed against the RBD of the SARS-CoV-2-S. This assay has a defined threshold for seropositivity of 0.8 U/mL, with a reported sensitivity of 98.8% and a specificity of 99.98%.^9^

### Surrogate SARS-CoV-2 Neutralization Tests (sVNTs)

Neutralization capacity against the ancestral SARS-CoV-2 virus and Omicron subvariants (BA.1, BA.2, and BA.4/5) was evaluated by using cPass SARS-CoV-2 neutralization antibody detection kit (GenScript, Singapore) at baseline, M1, and M3. This FDA-authorized assay is a semiquantitative, competitive enzyme-linked immunosorbent assay (ELISA) that measures the interaction between the angiotensin-converting enzyme 2 (ACE2) receptor and the RBD of the SARS-CoV-2 spike protein, providing a surrogate measure of neutralizing activity.^10^ In accordance with the manufacturer’s instructions, the presence of neutralizing antibodies is determined by an inhibition threshold of 30%.

### Pseudovirus Neutralization Tests (PVNTs)

To assess neutralization efficacy against emerging SARS-CoV2 variants following bivalent vaccination, we performed PVNTs against the ancestral virus and several Omicron subvariants, including BA.1, BF.7, B.2.75, BQ.1.1, and XBB.1.5 at baseline and M1 in 20 infection-naïve HD patients and 10 age-matched controls. Neutralizing antibody titers were quantified using a lentiviral pseudovirus system as previously prescribed.^11^ Complement-inactivated sera from vaccinees were serially diluted and incubated with 100 tissue culture infectious dose (TCID_50_) of SARS-CoV-2 S-expressing pseudovirus for 1 hour. The serum–virus mixture was then added to a HEK293 cell line stably overexpressing human ACE2 (HEK293-ACE_O/E_) in a seeding plate. After 18 to 24 hours of incubation, the infection rate was measured using a Nano-Glo Luciferase Assay System (Promega, Madison, WI). The resulting 50% pseudovirus neutralization titers (pVNT_50_) were calculated and subsequently converted into international units/milliliter (IU/mL) to facilitate comparability between studies.

#### SARS-CoV-2 specific interferon-γ (IFN-γ) release assay (IGRA)

Cell-mediated immune responses were evaluated using SARS-CoV-2 specific interferon-γ release assay, specifically the Covi-FERON ELISA (SD Biosensor, Suwon, Republic of Korea) at M1.^12^ This assay quantifies the IFN-γ production by T cells in response to SARS-CoV-2 antigens, providing a measure of cellular immunity. Whole blood samples (1 mL) from participants were distributed into each Covi-FERON tubes (nil tube, as negative control; mitogen tube, as positive control; and total SP tube, containing spike protein antigens derived from alpha, beta, gamma, delta, and omicron variants). Samples were incubated for 24 hours to stimulate T cell response. Following incubation, IFN-γ levels in plasma were quantified using an ELISA. Quality control and data interpretation were performed using ELISA Report Software (SD Biosensor), with a positive cut-off value as 0.25 IU/mL.

### Statistical Analysis

Data in parametric or nonparametric distribution were expressed as mean (± standard deviation [SD]) or median (interquartile range [IQR]), respectively. Continuous variables between groups were compared using *t* test for parametric data or Mann–Whitney *U* test for nonparametric data. We compared categorical variables using the chi-square test or the Fisher exact test, as appropriate. Comparisons of antibody titers (visualized as violin plots in Figure 1, Figure 2, Figure 3, Figure 4, Figure 5) were conducted using the Kruskal–Wallis test and Dunn’s post hoc test. The Holm–Bonferroni sequential correction method was applied to all pairwise comparisons to control for familywise error rates. To address multiple comparisons across groups and time points, we employed the Holm–Bonferroni sequential correction method. This approach provides robust control over the familywise error rate while offering improved statistical power compared with the traditional Bonferroni adjustment.^13^^,^^14^ To account for the repeated measurements nature of the data, we constructed multivariate linear mixed effect model to clarify the effect of independent variables on antibody level at various time points. Correlations between variables were analyzed using Spearman’s rank correlation coefficient. Statistical significance was set at a *P* value < 0.05 for all analyses. All statistical analyses were performed using SAS, version 9.4 (SAS Institute , Cary, NC, USA). Graphical representations were generated using R 4.4.0 software (R Foundation for Statistical Computing, Vienna, Austria).

**Figure 1:**
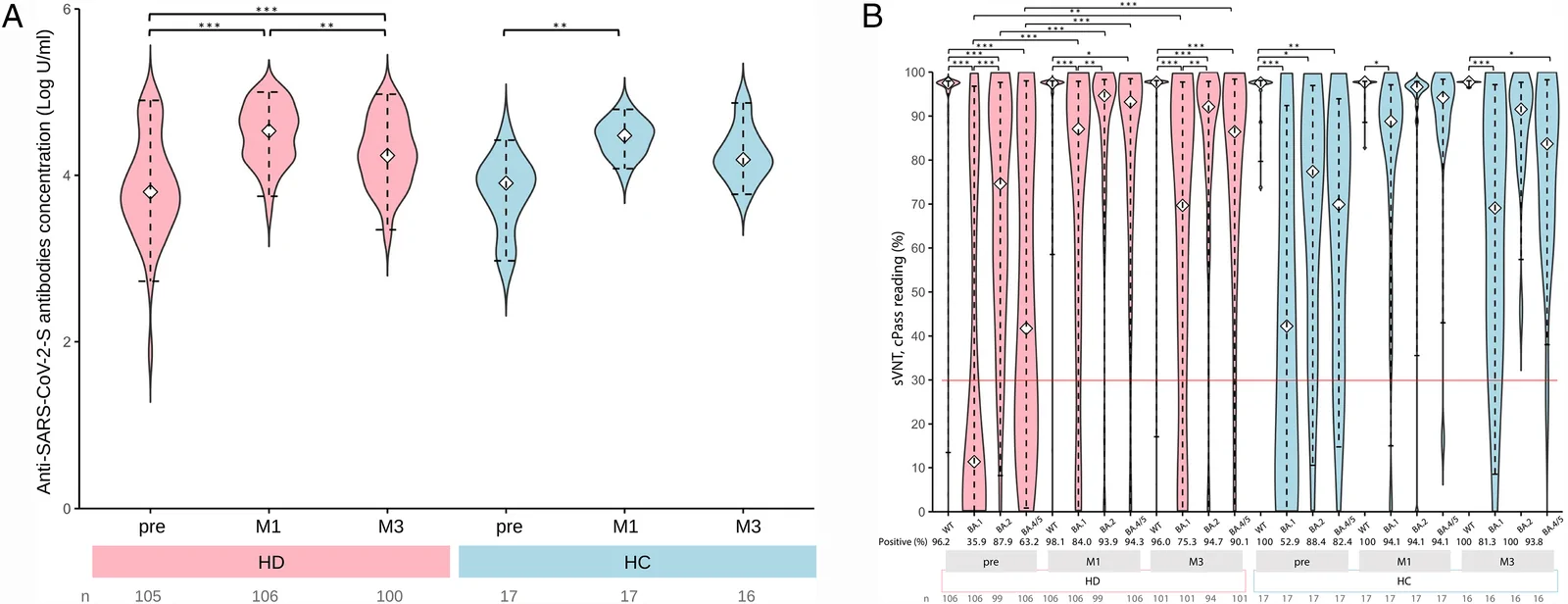
Trajectory of anti-SARS-CoV-2-S antibodies (A) and neutralizing antibodies against the ancestral virus, Omicron BA.1, BA.2 and BA.4/5 in surrogate neutralization tests (sVNT) (B) following bivalent vaccination in hemodialysis patients (HD) and health controls (HC). The horizontal red line in (B) indicates the positive threshold of sVNT (cPass reading ≥ 30%). The seropositive rates were listed, indicating proportion of participants with a cPass reading ≥ 30%. ∗*P* < 0.05, ∗∗*P* < 0.01, ∗∗∗*P* < 0.001. Abbreviations: Pre, before bivalent vaccination; M1, one month after vaccination; M3, three months after vaccination.

**Figure 2:**
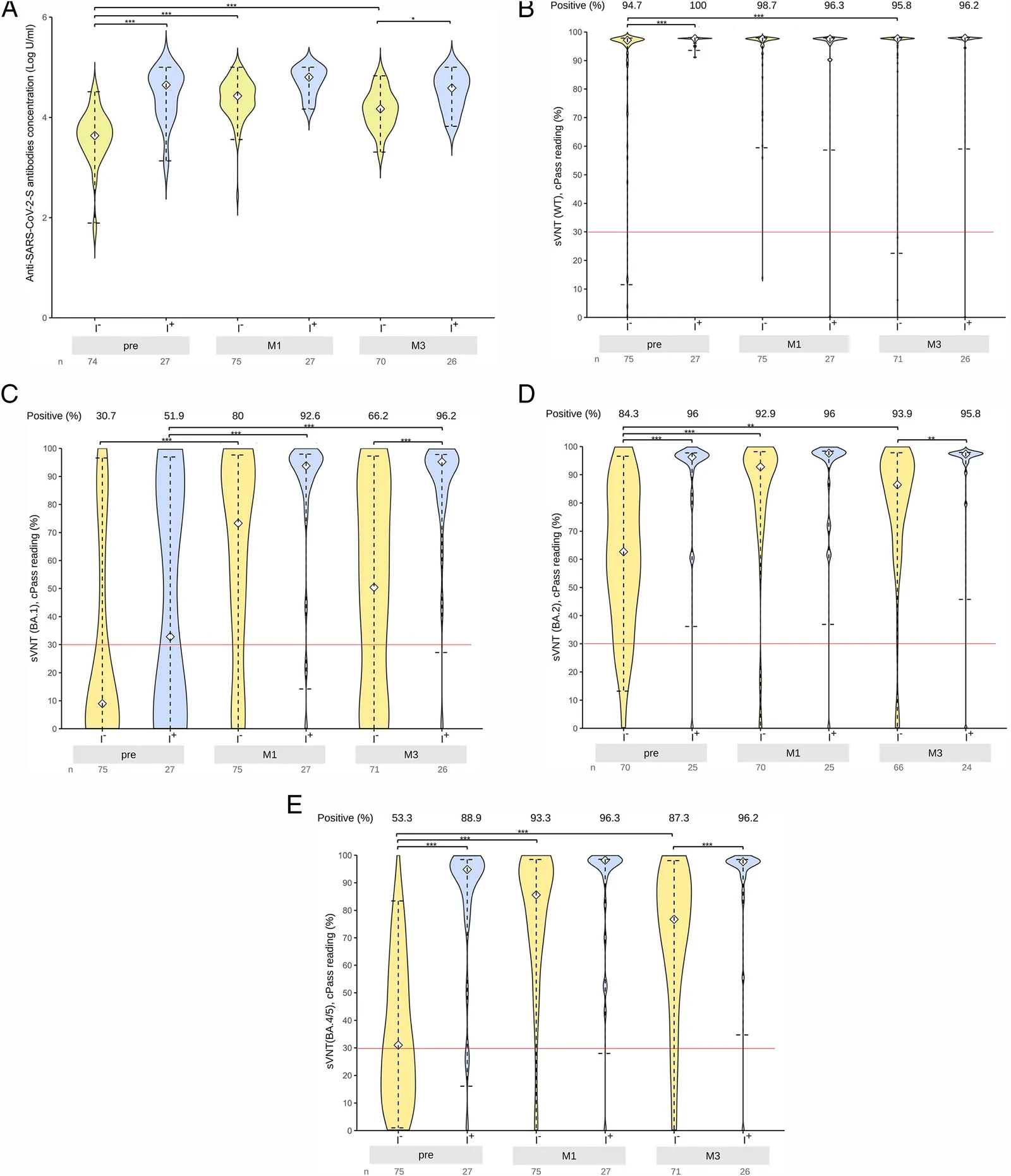
Trajectory of humoral immune responses in SARS-COV-2 infection-naïve (I^-^) and convalescent (I^+^) hemodialysis patients. (A) Anti-SARS-CoV-2-S antibodies. (B-D) sVNT against the ancestral virus, Omicron BA.1, BA.2, and BA.4/5. The horizontal red line in (B-D) indicates the positive threshold of sVNT (cPass reading ≥ 30%). The seropositive rates were listed, indicating proportion of participants with a cPass reading ≥ 30%. ∗*P* < 0.05, ∗∗*P* < 0.01, ∗∗∗*P* < 0.001. Abbreviations: Pre, before bivalent vaccination; M1, one month after vaccination; M3, three months after vaccination.

**Figure 3:**
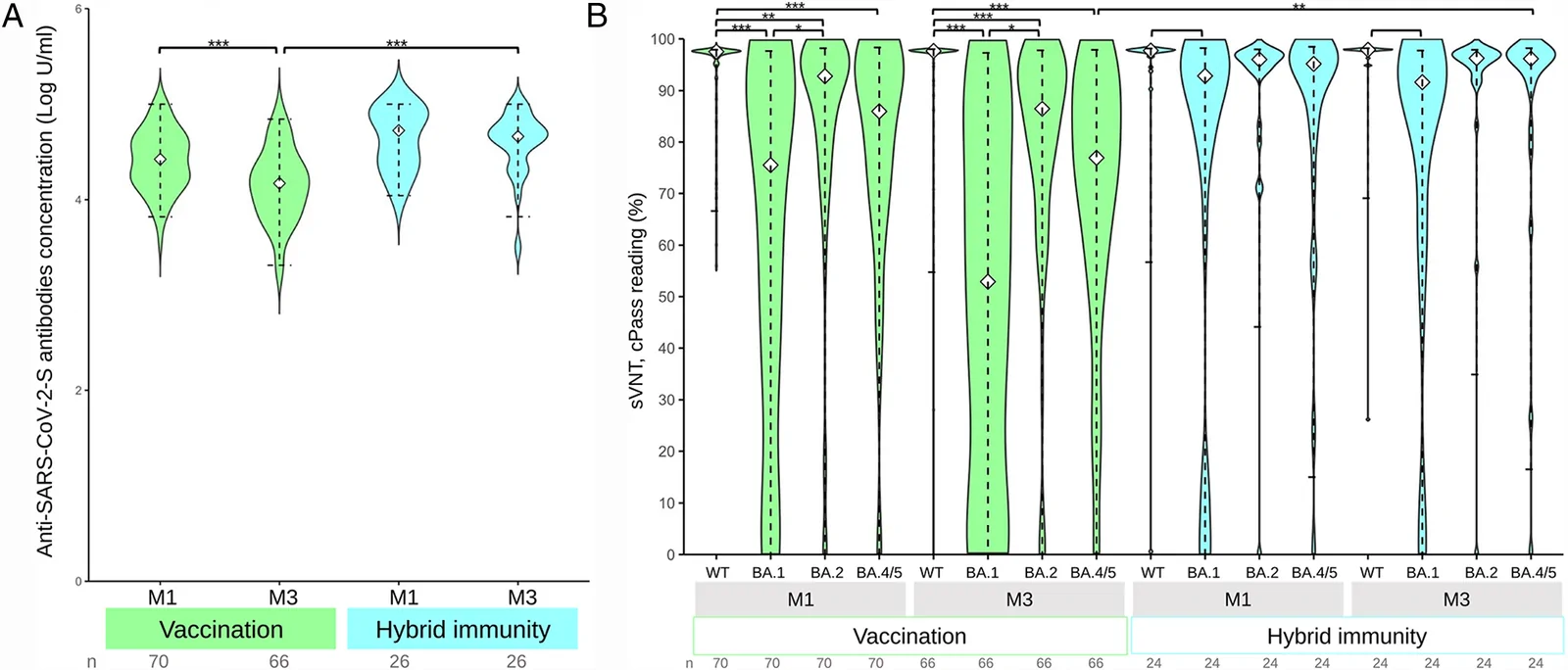
Anti-SARS-CoV-2 antibodies (A) and sVNT against the ancestral virus, Omicron BA.1, BA.2, and BA.4/5 (B) at 1 and 3 months following the fifth immunization in HD patients with pure vaccination or hybrid immunity (4 doses of vaccine plus one infection). Abbreviations: M1, one month after vaccination; M3, three months after vaccination.

**Figure 4:**
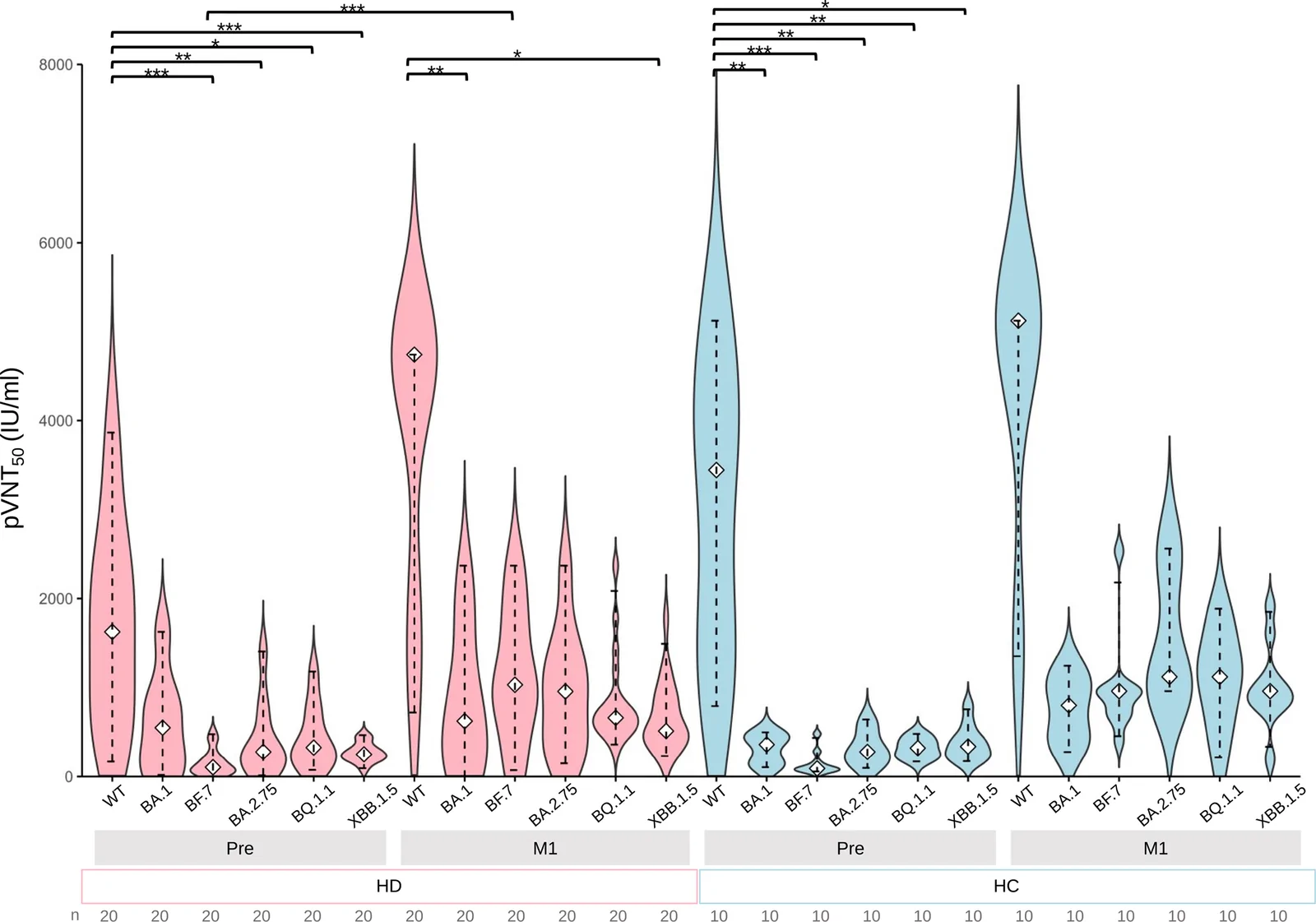
Pseudovirus neutralization tests against the ancestral virus, Omicron BA.1, BF.7, BA.2.75, BQ.1.1, and XBB.1.5 before and one month (M1) after bivalent vaccination in hemodialysis patients (HD) and health controls (HC). ∗ *P* < 0.05, ∗∗ *P* < 0.01, ∗∗∗ *P* < 0.001, ∗∗∗∗ *P* < 0.0001.

**Figure 5:**
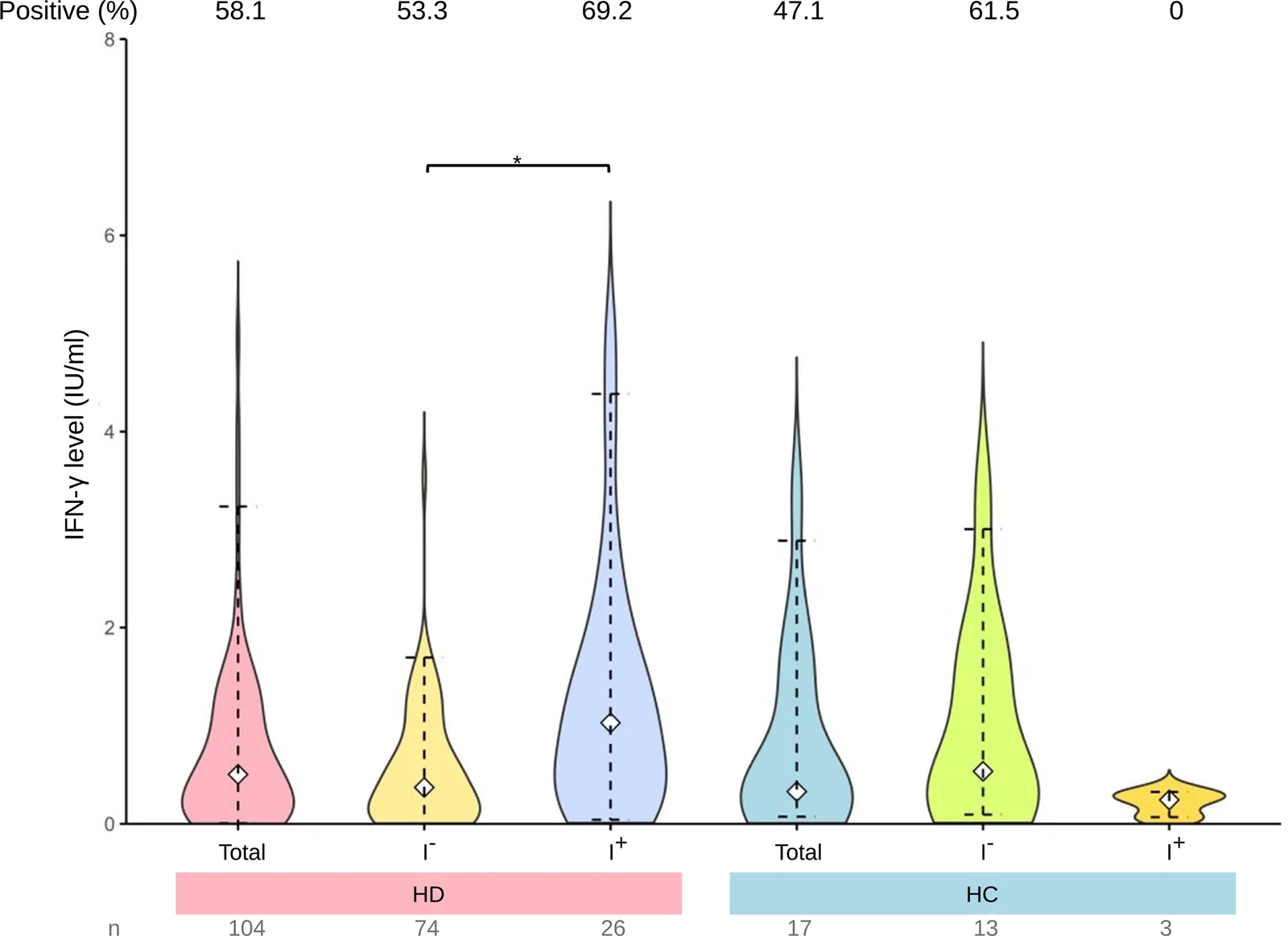
Interferon-γ concentrations in Covi-FERON at one month after bivalent vaccination in hemodialysis patients (HD) and health controls (HC), stratified by previous SARS-CoV-2 infection (infection naïve, I^-^; convalescent, I^+^).∗*P* < 0.05.

## Results

### Study Cohort and Baseline Characteristics

We enrolled 106 HD patients (median age 66 years, 51% female and median dialysis vintage 5.3 years) who received bivalent mRNA boosters. The majority received Spikevax bivalent Original/Omicron BA.1 (89.6%), and 10.4% received Spikevax bivalent Original/Omicron BA.4-5. Most patients (93.4%) had previously received 4 doses of SARS-CoV-2 vaccines with 25.5% having experienced previous breakthrough infection. The median time interval between the last SARS-CoV-2 antigen exposure, either vaccination or documented infection, was 147 days. The control group comprised 17 individuals (median age 67 years), with 70.6% having received 4 SARS-CoV-2 vaccinations and 24.9% having received 3 doses. One control participant has a previous asymptomatic SARS-CoV-2 infection. Four initially infection-naïve HD participants developed asymptomatic infections within three months postvaccination, as indicated by anti-SARS-CoV-2 N antibodies. During the 9-month follow-up, 17 HD patients (median age 70 years) experienced symptomatic COVID-19, diagnosed 30-237 days (median 172 days) after bivalent vaccination. One COVID-19-related death occurred in this group. Detailed baseline characteristics are present in Table 1.

**Table 1:** Baseline Characteristics of the Study Cohort Receiving Bivalent SARS-CoV2 Vaccination With and Without Previous Breakthrough Infection.

	Total Dialysis Patients (N = 106)	Patients Received Bivalent mRNA Booster (Ancestral Virus + Omicron BA.1 or BA.4/5)	
		No Previous Breakthrough Infection (N = 79)	Previous Breakthrough Infection^a^ (N = 27)
Age, year	66 (60, 72)	66 (60, 72)	64 (46, 74)
Female, N (%)	51 (48.11)	39 (49.37)	12 (44.44)
Dialysis vintage, year	5.3 (3.71, 7.83)	5.08 (3.75, 7.83)	6.08 (3.41, 7.83)
Type of bivalent vaccine			
BA.1	95	76	19
BA.4/5	11	3	8
Dose of previous SARS-CoV-2 vaccination			
Two	2	2	0
Three	5	3	2
Four	99	74	25
Time since last immunization/infection^b^	147 (147, 147)	147 (147, 147)	110.5 (96, 134.3)
BMI, kg/m^2^	22.72 (20.11, 25.63)	23.01 (20.74, 25.63)	22.31 (19.61, 26.27)
Kt/V	1.79 (1.69, 1.96)	1.79 (1.69, 1.98)	1.8 (1.65, 1.92)
Immunosuppressant use	2 (1.89)	1 (1.27)	1 (3.7)
Comorbid condition, N (%)			
Diabetes mellitus	58 (54.72)	47 (59.49)	11 (40.74)
Hypertension	98 (92.45)	75 (94.94)	23 (85.19)
Heart failure	24 (22.64)	20 (25.32)	4 (14.81)
CVA	6 (5.66)	5 (6.33)	1 (3.7)
COPD	2 (1.89)	1 (1.27)	1 (3.7)
MI	7 (6.6)	5 (6.33)	2 (7.41)
Malignancy	24 (22.64)	17 (21.52)	7 (25.93)

Abbreviations: BMI, Body mass index; CVA, cerebrovascular accident; COPD, chronic pulmonary obstructive disease; MI, myocardial infarction.

a The dominant SARS-COV-2 variants were Omicron BA.2 and subsequent BA.5 before enrollment.

b Excluded the 9 participants who had asymptomatic infection (defined as positive anti-SRAS-CoV-2 N antibodies any time before the bivalent vaccination) because the time intervals between last infection and the bivalent vaccination were uncertain.

### Evaluation of Humoral Immunity by Measuring Anti-SARS-CoV-2-S RBD Antibodies and sVNT Following Bivalent Boosters

Anti-SARS-CoV-2-S RBD antibodies concentrations in HD patients exhibited a fourfold increase 1 month after bivalent boosters administration, followed by a 50% decrease 2 months later (8,128, 33,884, and 17,378 U/mL at pre-, M1, and M3, respectively). No significant differences in antibodies titers was observed between HD patients and controls at each time point (Fig 1A). After excluding participants with anti-N antibodies at M3 and stratifying patients based on prior SARS-CoV-2 infection, we found patients with previous infection demonstrated high baseline Anti-SARS-CoV-2-S RBD antibodies levels before bivalent vaccination (Fig 2A). The titers increased significantly following the bivalent booster in infection-naïve patients, but not in patients with previous infection.

sVNT showed significant increases in neutralization against Omicron sublineages BA.1, BA.2, and BA.4/5 postbivalent boosters, sustained for 3 months. Neutralization against the ancestral virus remained high throughout. Neutralization against the ancestral virus consistently exceeded that of Omicron sublineages, with BA.1 showing the lowest. No significant differences were observed between HD patients and controls (Fig 1B). Seropositive rates for ancestral virus, BA.1, BA.2, and BA.4/5 at baseline, M1, and M3 were 96.2%, 35.9%, 87.9%, 63.2%; 98.1%, 84%, 93.9%, 94.3%; and 96%, 75.3%, 94.7%, 90.1%, respectively. Anti-SARS-CoV-2-S RBD antibodies titers and cPass readings against the ancestral virus and omicron sublineages demonstrate correlation at each time point (Fig S2).

Baseline neutralization against the ancestral virus, BA.2, and BA.4/5 were lower in the infection-naïve versus previously infected group. The latter showed no significant difference between neutralization against the ancestral virus and BA.2 (*P* = 0.24) or between BA.2 and BA.4/5 (*P* = 0.25) before bivalent vaccination, align with prevalent strains (BA.2 and subsequently BA.5) in Taiwan during the study period.^15^^,^^16^ Postbivalent vaccination, infection-naïve patients exhibits significant increase in the neutralization against BA.1, BA.2, and BA.4/5, whereas previously infected patients showed increased neutralization only against BA.1. Despite at 3 months postvaccination, the neutralization against Omicron sublineages in the infection-naïve group remained weaker than in the previously infected group (Fig 2C-E).

Stratifying participants by antigen exposure showed that HD patients with three prior vaccinations plus bivalent vaccination (number of exposures = 4) had low pre-vaccination antibody levels(median anti-S: 60 U/mL, median cPass: <30%) and poor neutralization against Omicron sublineages after bivalent booster (median cPass at M1: <30%), indicating inadequate protection. Conversely, patients with 6 antigen exposures (1 natural infection and 5 vaccinations, including 1 bivalent vaccine) demonstrated robust and sustained humoral immunity (anti-SARS-COV-2-S antibodies 63,096 U/mL at M1; sVNT > 95% against all strains at M3; Fig S3A-E).

Comparing humoral responses in hemodialysis patients with 5 antigen exposures, we analyzed those with 5 vaccinations (n = 70) versus 4 vaccinations plus 1 infection (n = 26, including 12 without bivalent boosters from our previous study). The hybrid immunity group exhibited significantly higher anti-SARS-CoV-2-S antibody titers (42,658 vs 14,791 U/mL at M3, *P* < 0.001) and tended to show superior neutralizing capacity, with cPass readings above 90% against all strains at M1 and M3 (Fig 3). This suggests hybrid immunity confers stronger and more durable humoral responses than vaccination alone.

### Assessment of Neutralization Capacity of Bivalent Vaccine Against New Emerging Omicron Subvariants

One month postvaccination, the neutralization increased 1.14-9.94 times, with BF.7 showing significant improvement. The median neutralizing antibody titers to BA.1, BF.7, B.2.75, BQ.1.1, and XBB.1.5 were 7.6-, 4.6-, 5.0-, 7.2-, and 9.3-fold lower than ancestral virus. No significant difference between HD patients and controls were observed (Fig 4). At M1, the cPass reading against BA.1, BA.2, and BA.4/5 were correlated to pVNT_50_ against BA.1, BF.7, and BA.2.75 (Fig S2).

### Cellular Immunity Following Bivalent Boosters

At M1, Covi-FERON showed positive cellular immunity specific to SARS-CoV2 in 58.1% of HD patients versus 47.1% of controls. The median IFN-γ level were 0.50 and 0.33 IU/mL in HD and control groups, respectively, without significant difference (Fig 5). Previously infected patients had higher IFN-γ than infection-naïve patients (1.03 vs 0.37 IU/mL, *P* = 0.05). Cellular responses were present in 53.3% of infection-naïve and 69.2% of previously-infected patients. IFN-γ levels did not correlate with any humoral outcome, including anti-SARS-CoV-2-S antibodies, sVNT, or PVNT (Fig S2).

### Predictors of Humoral Responses Following Bivalent Vaccinations

Multivariate linear mixed effect model showed significantly higher anti-SARS-CoV-2-S RBD antibodies and neutralization against omicron BA.1, BA.2, and BA.4/5 in sVNT at month 1 and 3 compared with baseline. A greater number of antigen exposures (either infection or vaccination) correlated to higher anti-SARS-CoV-2-S RBD antibodies and neutralization against the ancestral virus, but not with neutralization against omicron sublineages or IFN-γ levels in Covi-FERON. Notably, patients with previous infection demonstrated lower neutralizing capacity against the ancestral virus. Advanced age was associated with weaker cellular responses at M1 (Table 2).

**Table 2:** Parameters Associated with Binding Antibody Titer Against Spike Receptor-Binding Domain, Neutralizing Antibodies Against 4 Strains of SARS-Cov-2 and Interferon-Releasing Assay in Hemodialysis Patients Following the Bivalent SARS-Cov-2 Vaccination.

Parameter	Anti-SARS-CoV-2-S Antibody Titer		Surrogate Virus Neutralization Test (cPass Reading)								Covi-FERON (IFN-γ Concentration)	
			Ancestral Virus		Omicron BA.1		Omicron BA.2		Omicron BA.4/5		Estimate (standard error)	*P* value
	Estimate (standard error)	*P* value	Estimate (standard error)	*P* value	Estimate (standard error)	*P* value	Estimate (standard error)	*P* value	Estimate (standard error)	*P* value		
Time (Reference=pre)												
M1	1.48 (0.08)	<0.001	2.01 (1.98)	0.31	59.71 (7.12)	<0.001	16.68 (1.64)	<0.001	32.28 (2.13)	<0.001	n.a.	n.a.
M3	0.96 (0.08)	<0.001	2.21 (2.01)	0.27	51.28 (7.22)	<0.001	14.84 (1.67)	<0.001	28.22 (2.17)	<0.001	n.a.	n.a.
Number of immunizations	1.24 (0.26)	<0.001	13.24 (3.72)	<0.001	13.94 (11.07)	0.21	0.88 (20.03)	0.97	11.08 (6.92)	0.11	0.08 (0.18)	0.65
Infection before bivalent vaccines	–0.13 (0.33)	0.7	–11.81 (4.72)	0.01	9.19 (14.06)	0.51	13.38 (19.60)	0.50	12.79 (8.77)	0.15	0.25 (0.23)	0.28
Age	–0.01 (0.01)	0.30	-0.09 (0.10)	0.40	–0.16 (0.31)	0.61	–0.12 (0.22)	0.60	–0.32 (0.19)	0.10	–0.01 (0.01)	0.04
DM	0.21 (0.18)	0.26	–3.18 (2.62)	0.23	–1.33 (7.81)	0.87	–1.91 (5.40)	0.72	0.73 (4.86)	0.88	–0.03 (0.13)	0.82

Abbreviations: M1, 1 month after bivalent vaccination; M3, 3 months after bivalent vaccination; DM, diabetes mellitus.

### Immune Responses in Patients with Breakthrough Infection After Bivalent Vaccination

During the 9-month follow-up, seventeen HD patients developed symptomatic COVID-19 after bivalent vaccination. At baseline, these patients exhibited lower anti-SARS-CoV-2-S antibodies (4281 vs 8128 U/mL) and reduced neutralization against Omicron BA.1, BA.2, and BA.4/5 (cPass: 12.5%, 54.5%, 23.5% vs 35.9%, 87.9%, 63.2%, respectively) compared with the overall cohort. At 1 month postvaccination, their antibody titers (20464 vs 33884 U/mL) and neutralizing capacity against BA.1, BA.2, and BA.4/5 (cPass: 62.7%, 88.3%, 79.6% vs 84%, 93.9%, 94.3%, respectively) remained lower. However, cellular responses were comparable between groups (IFN-γ: 0.504 vs 0.50 IU/mL). These findings suggest that lower humoral responses may predispose patients to breakthrough infection.

## Discussion

This observation cohort study investigated the immune responses following bivalent SARS-CoV-2 vaccination in maintenance hemodialysis patients, 25% of whom had prior SARS-CoV-2 infection and 90% had received 4 doses of monovalent vaccines. Following bivalent vaccination (containing mRNA from ancestral virus and Omicron BA.1 or BA.4/5), patients exhibited robust humoral immune responses. The anti-SARS-CoV-2-S antibodies titers increased 4-fold compared with the baseline level. sVNT showed significantly enhanced neutralization against omicron sublineages BA.1, BA.2, and BA.4/5, with over 80% of participants developing neutralizing antibodies. PVNT even showed a 9.9-fold increase in neutralization against the new emerging omicron BF.7 variant. In contrast, cellular responses specific to SARS-CoV2 quantified using IGRA 1 month after the bivalent vaccination were less pronounced, with only 60% patients had positive cellular responses. Notably, the vaccine-induced humoral and cellular responses in hemodialysis patients were comparable to those observed in healthy controls.

In general population, although bivalent BA.1 or BA.4/5 boosts induce neutralizing antibody against subvariants containing in vaccines, both of them provide broad cross-protection against other omicron subvariants, and the neutralization against BQ.1.1 and XBB.1 were comparable.^17^^,^^18^ In the existing literature, information about immune responses following bivalent omicron BA.1 vaccination in dialysis patients is scant. The immune responses in our cohort following bivalent vaccination are similar to results following bivalent BA.4/5 in HD patients in other studies. Bivalent boosters can significantly increase binding antibodies further, which correlate with neutralizing antibody titers against omicron subvariants.^19^, ^20^, ^21^ Although nearly all patients were able to neutralize the ancestral virus before bivalent vaccination, the seropositive rates of neutralizing antibodies against BA.1, BA.2, or BA.4/5 remained only ∼50%, which increased to 75%-100% after bivalent boosters, depending on the number of immunization and proportion with previous SARS-COV-2 in enrolled participants.^19^^,^^20^^,^^22^ The enhancement of humoral responses were more obvious in participants without previous SARS-CoV-2 infection than convalescents, in line with results from studies in HD patients or general population.^17^^,^^21^^,^^23^^,^^24^ In our study, the patients with previous infection which are mainly from Omicron BA.2 and BA.5 had significant increase only at neutralization against BA.1 following bivalent boosts.^15^^,^^16^ In multivariate linear mixed effect model, after adjusting variables including number of antigen exposure, infection before bivalent was associated with less neutralization against the ancestral virus. It was demonstrated that the neutralization against the ancestral virus following three vaccinations is comparable in general populations with and without Omicron infection.^25^ In other words, Omicron infection cannot boost neutralizing antibodies against the ancestral virus. Thus, the previous omicron infection in our cohort is reasonably associated with less neutralizing capacity against the ancestral virus under the premise of the same number of antigen exposure.

The breadth of vaccine-induced humoral response is a crucial parameter in evaluating vaccine efficacy. Our data showed a 1.14-9.94-fold increase in neutralizing titers against Omicron sublineages BF.7, BA.2.75, BQ.1.1, and XBB.1.5 in pseudovirus neutralization tests, although statistical significance was only achieved in BF.7, possibly because of limited sample size. These findings align with other studies in health individuals and dialysis patients, in which bivalent BA.1 or BA.4/5 mRNA vaccines similarly broaden neutralization against newer omicron subvariants with titers decreasing in the order of BF.7, BA.2.75, BQ.1.1, and XBB.1 as observed in our results.^26^, ^27^, ^28^ A vaccine effectiveness study in the general population associated the bivalent BA.4/5 mRNA boosters with lower risks of infection or severe disease with BQ.1–BQ.1.1 and XBB–XBB.1.5.^29^ Our results reinforce the evidence supporting bivalent mRNA boosters in the HD population, demonstrating their capacity the breadth of humoral responses and, consequently, improve clinical protection, even when the circulating subvariants differ from those originally targeted by the vaccines.

In contrast to robust humoral responses, bivalents vaccination did not significantly enhance cellular immunity. Forty percent HD patients showed no positive reaction to SARS-CoV-2 specific antigen in Covi-FERON despite most participants having exposed to SARS-CoV-2 antigens 5 to 6 times, including a bivalent boost and in some cases SARS-CoV-2 infection. This suboptimal cellular responses aligns with other studies, potentially relate to increased PD-1 and LAG-3 expressions in T cells following repeated mRNA vaccination, indicating immune tolerance.^3^^,^^30^ A longitudinal study using ancestral strain-stimulated ELISpot assay in an HD cohort observed a decline in positive cellular response rates from 87.5% after the fourth dose to 58.6% following a subsequent bivalent BA.4/5 booster.^31^ Conversely, another study showed increased SARS-CoV-2 specific CD4^+^ and CD8^+^ cells and cytokine expression against both the ancestral and BA.4/5 strains following the bivalent BA. 4/5 vaccination in 35 HD patients (74.1% and 97.1% with CD4^+^ response, 60% and 89% with CD8^+^ response to BA.4/5 pre- and postboost, respectively).^22^ Although we did not measure cellular responses immediately before bivalent vaccination, our observed response rate is lower than previous measurements in this cohort using Covi-FERON (76.8% and 61.6% response to the ancestral virus or alpha and beta or gamma, respectively, before the fourth dose; 87.9% and 81.8%, respectively, after the fourth dose).^8^ Given the heightened susceptibility to apoptosis and dysregulation in activated and memory T and B cell populations in dialysis patients, further investigation is warranted to determine optimal COVID-19 vaccination strategies that enhance the magnitude, durability, and breadth of cellular immunity in this vulnerable population.^32^^,^^33^

Our study presents several notable strengths. It provides comprehensive assessment of vaccine responses in hemodialysis patients, examining both humoral and cellular immunity against multiple Omicron variants. The antibodies binding to structural proteins (eg, anti-SARS-CoV-2-S RBD antibodies in this study) are rapid, widely used initial assessments of humoral response, whereas neutralization tests directly measured capacity of antibodies to inhibit viral binding/entry of specific variants and correlate better with protection to breakthrough infection.^34^^,^^35^ Cellular responses following infection/vaccination last longer and remain cross-protection to variants compared with humoral immunity.^36^^,^^37^ With enrollment of healthy controls and systematically longitudinal follow-up, we documented detailed vaccination and infection history using rapid antigen testing and antibody measurements, enabling precise evaluation of vaccine-induced immunity in a real-world clinical setting and offer valuable insights into the trajectory of immune responses in hemodialysis patients.

As a limitation in this study, the results were derived from a limited sample of hemodialysis patients, which suggests that our findings may not be fully generalizable to the entire dialysis population, particularly those undergoing peritoneal dialysis or hemodialysis patients of races other than Asia. However, our sample size is relatively large compared with previous studies conducted in dialysis patients, lending additional weight to the validity of our conclusions.^19^, ^20^, ^21^, ^22^^,^^28^ Another limitation is the restricted sample size (n = 30) for neutralization assays against new emerging Omicron variants, necessitated by the labor-intensive and time-consuming nature of PVNTs. This constraint potentially increases the risk of type II errors in comparative analyses because of insufficient statistical power. Consequently, the interpretation of these specific results warrants caution. A third limitation of our study is the heterogeneity in bivalent vaccination received by participants. The majority (89.6%) received the Original/Omicron BA.1 formulation, thus our findings predominantly reflect its effects on vaccine-induced immunity. However, our preliminary analysis showed comparable humoral and cellular immunity responses between the two vaccine formulations (data not shown). This similarity supported the decision to combine the two vaccine groups for final analyses, which is unlikely to significantly bias the main conclusions of our study.

In conclusion, bivalent vaccines containing Omicron BA.1 or BA.4/5 mRNA enhanced humoral immunity and broaden neutralization against newer Omicron variants in HD patients, particularly in infection-naïve individuals. Nevertheless, the absence of enhanced cellular immunity underscores the necessity for further modifications to optimize immune response comprehensively.
