# Supplementary material for: Humoral and Cellular Immune Responses Following Bivalent SARS-CoV-2 mRNA Vaccines in Patients Undergoing Hemodialysis: A Prospective Cohort Study
Source: Kidney Med. 2026 Jul 23;8(10):101480. doi: 10.1016/j.xkme.2026.101480 (PMC13625874; doi:10.1016/j.xkme.2026.101480)
Supplement: Supplementary file 1 [file mmc1.pdf]

Supplementary Material

Humoral and Cellular Immune Responses Following Bivalent SARS-CoV-2 mRNA Vaccines in Patients Undergoing Hemodialysis: A Prospective Cohort Study

Contents

Supplementary Figure 1. Schedule of blood sampling and various assessments for immune responses in this study. - Page 2

Supplementary figure 2. Heatmaps representing correlations between immunity outcomes at baseline, one and three months following bivalent vaccination in HD patients. - Page 3

Supplementary Figure 3. Humoral immunity following bivalent vaccination in hemodialysis patients (HD) and health controls (HC) stratified by number of immunizations. (A) Anti-SARS-CoV-2 antibodies, (B)-(E) surrogate viral neutralization against the ancestral virus, Omicron BA.1, BA.2 and BA.4/5. - Page 4

Figure S1. Schedule of blood sampling and various assessments for immune responses in this study.

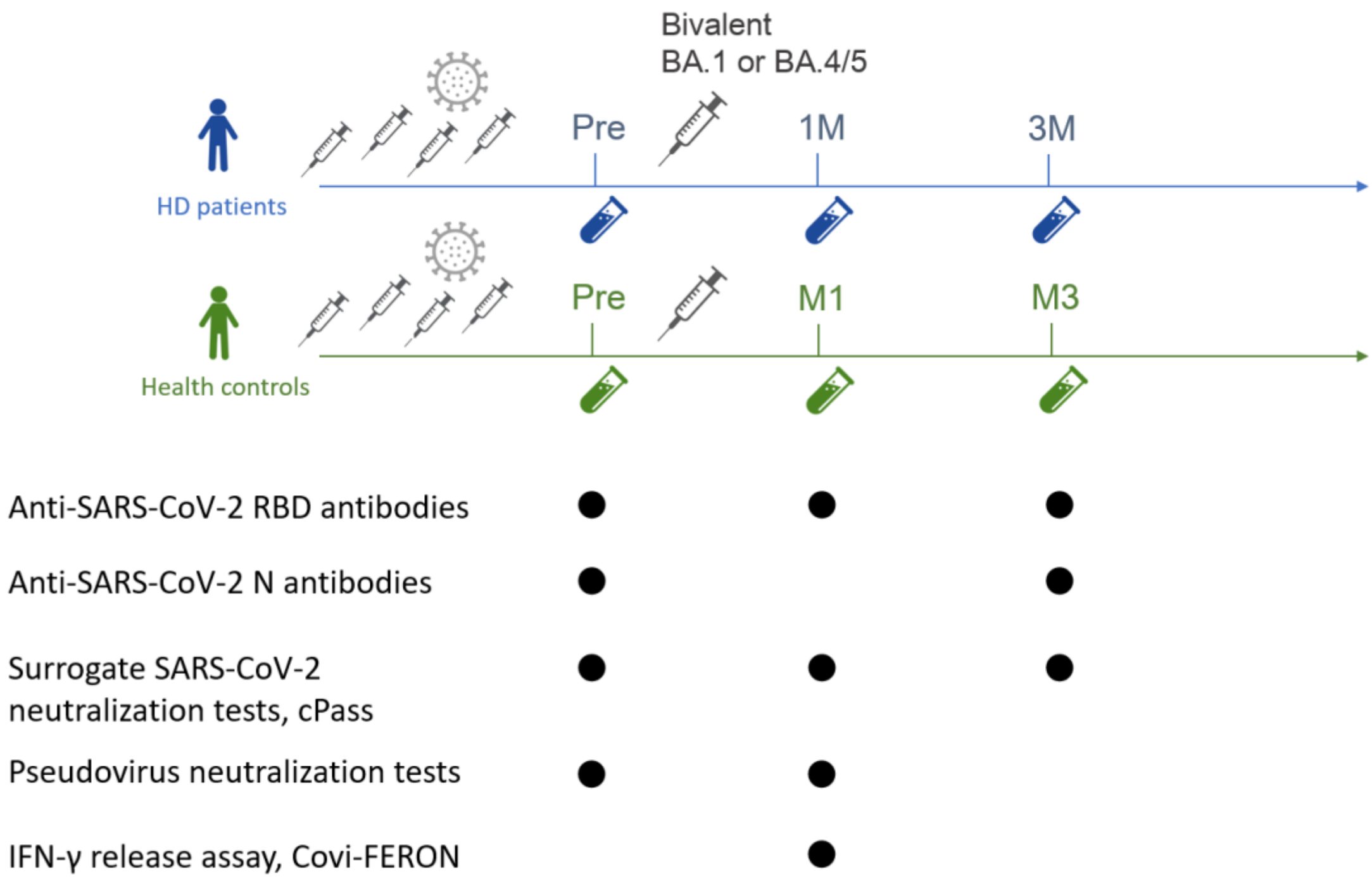

Figure S2. Heatmaps representing correlations between immunity outcomes at baseline, one and three months following bivalent vaccination in HD patients

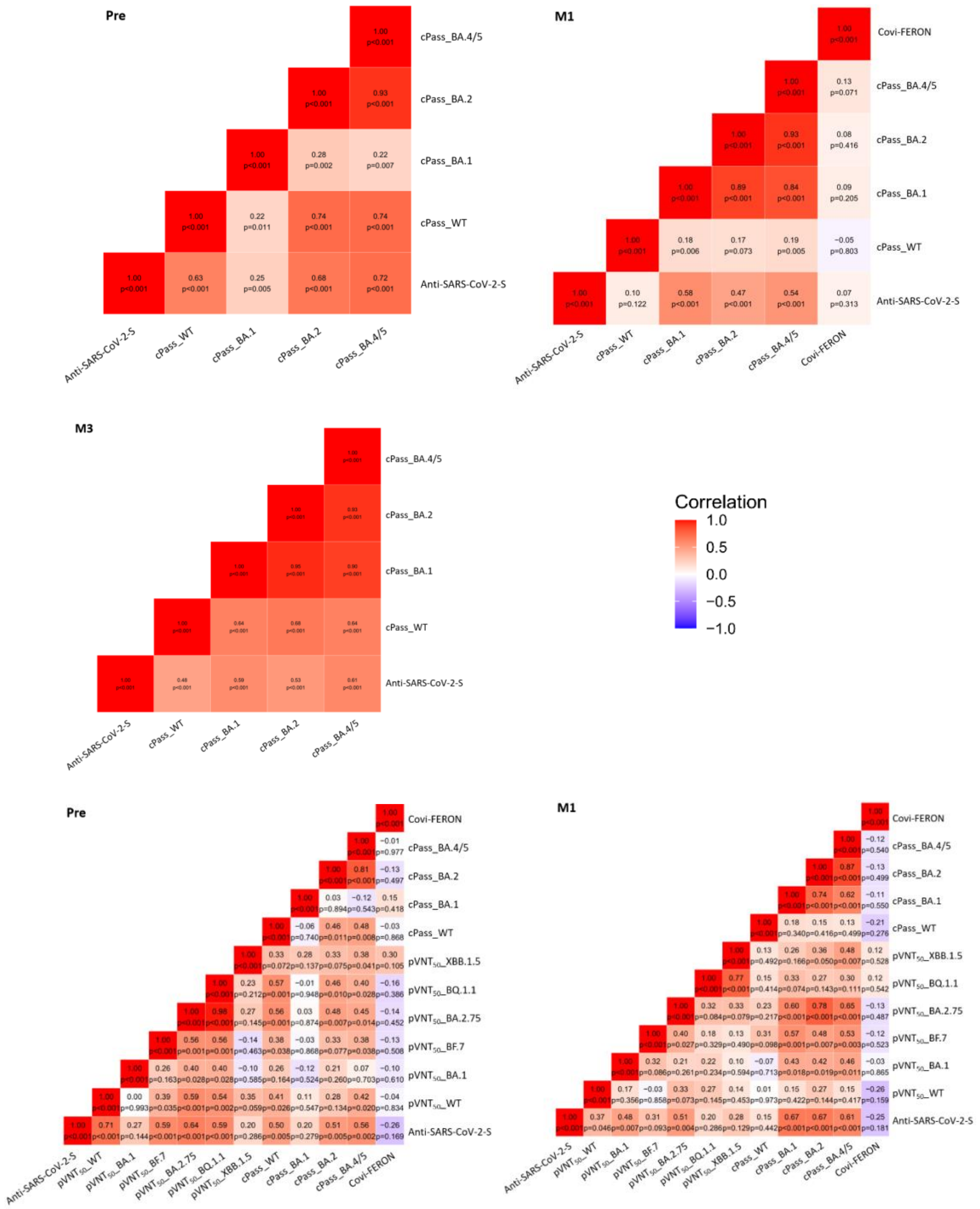

Figure 3. Humoral immunity following bivalent vaccination in hemodialysis patients (HD) and health controls (HC) stratified by number of immunizations. (A) Anti-SARS-CoV-2 antibodies, (B)-(E) surrogate viral neutralization against the ancestral virus, Omicron BA.1, BA.2 and BA.4/5.

(A)

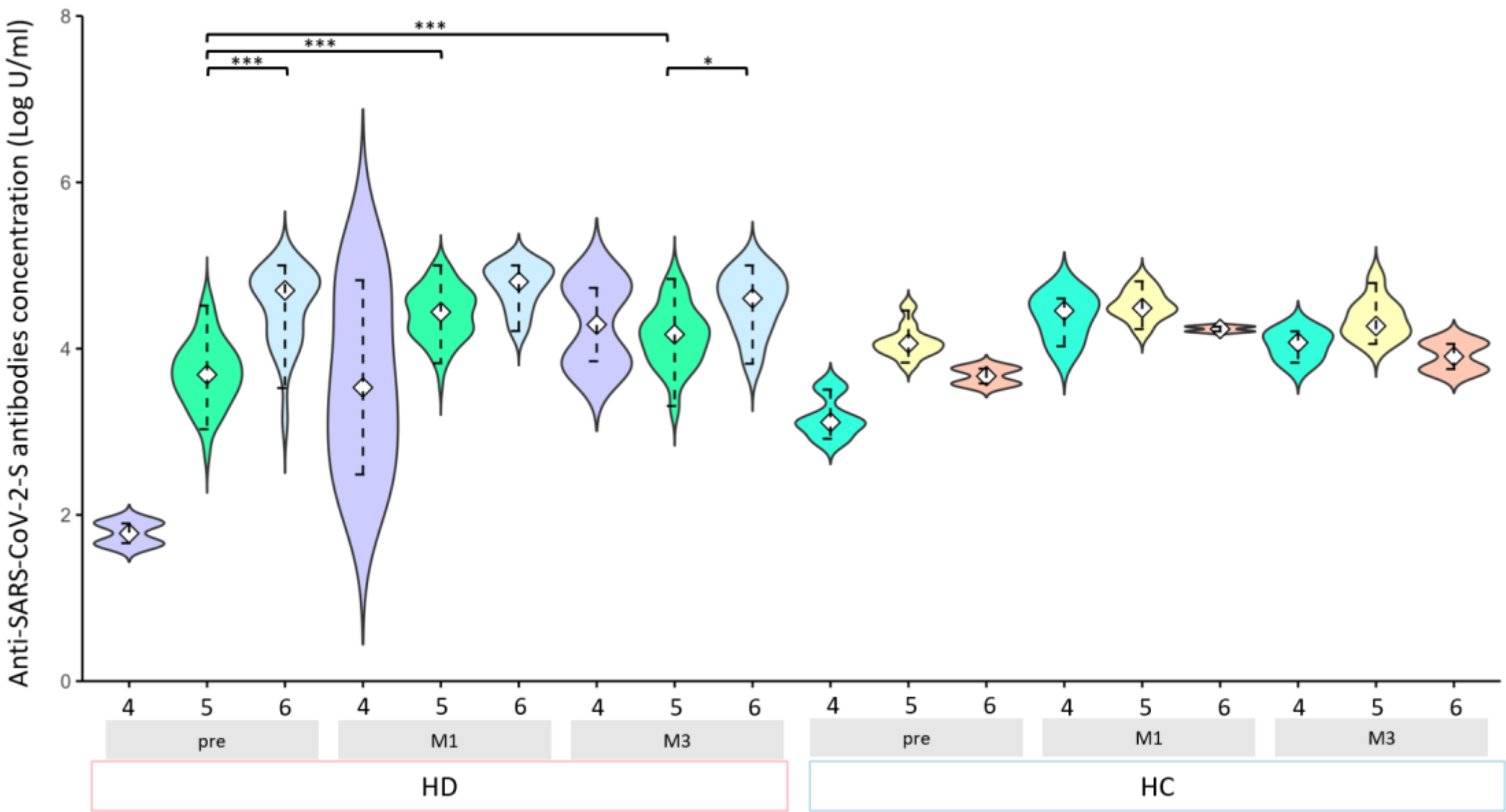

(B)

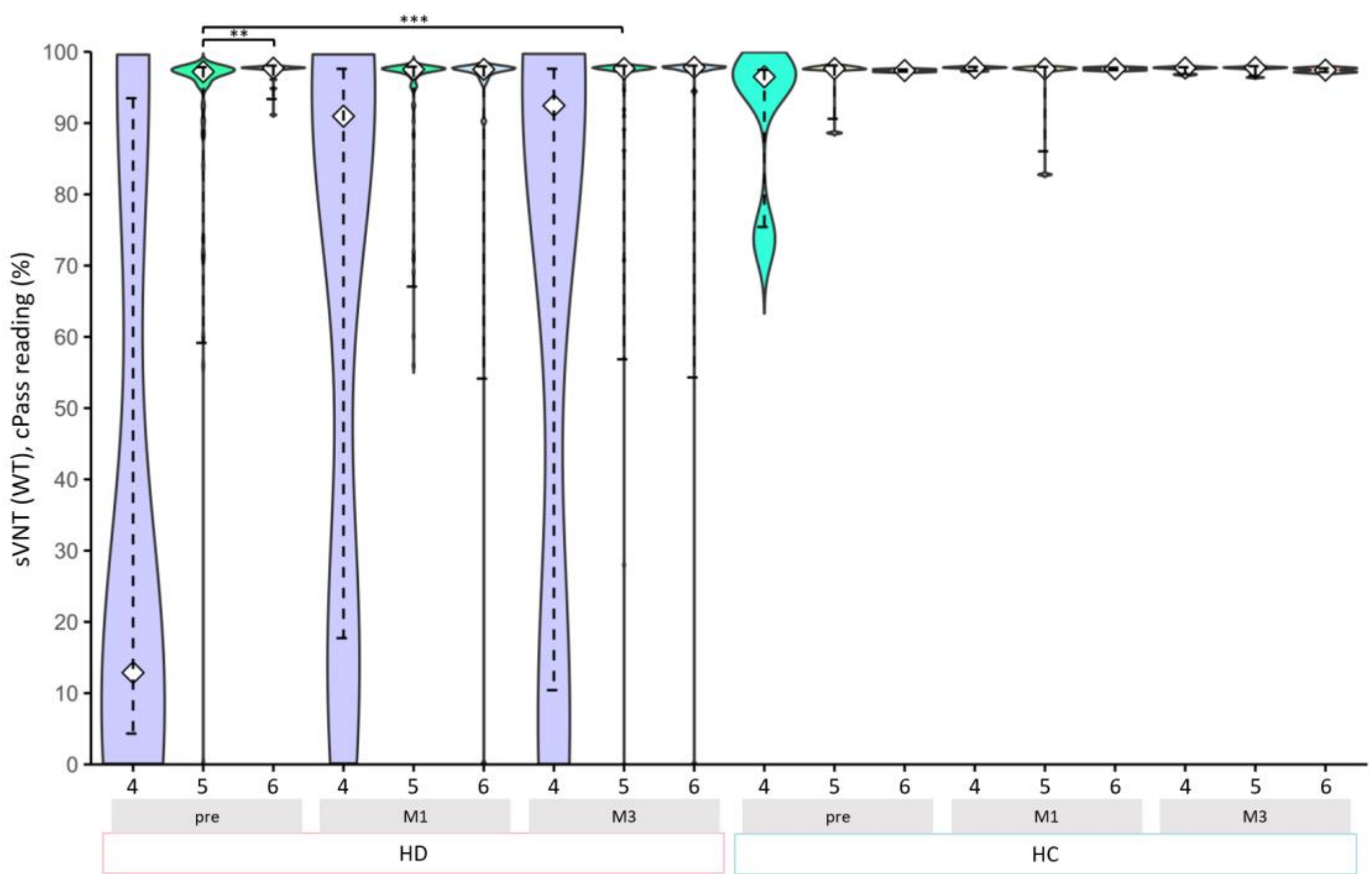

(C)

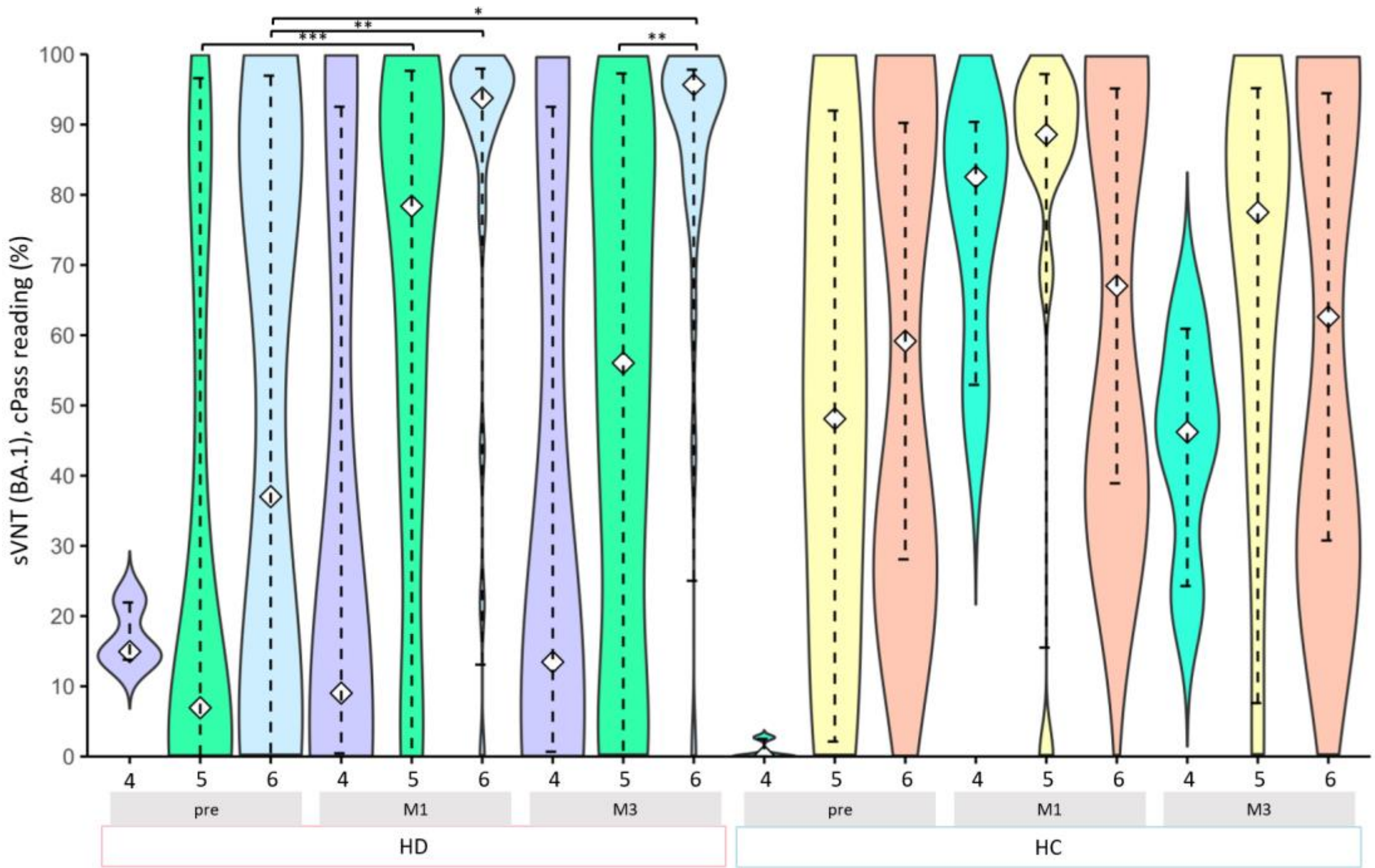
sVNT (BA.1), cPass reading (%)
100
90
80
70
60
50
40
30
20
10
0
***
**
*
**
4
5
6
4
5
6
4
5
6
4
5
6
4
5
6
4
5
6
pre
M1
M3
pre
M1
M3
HD
HC

(D)

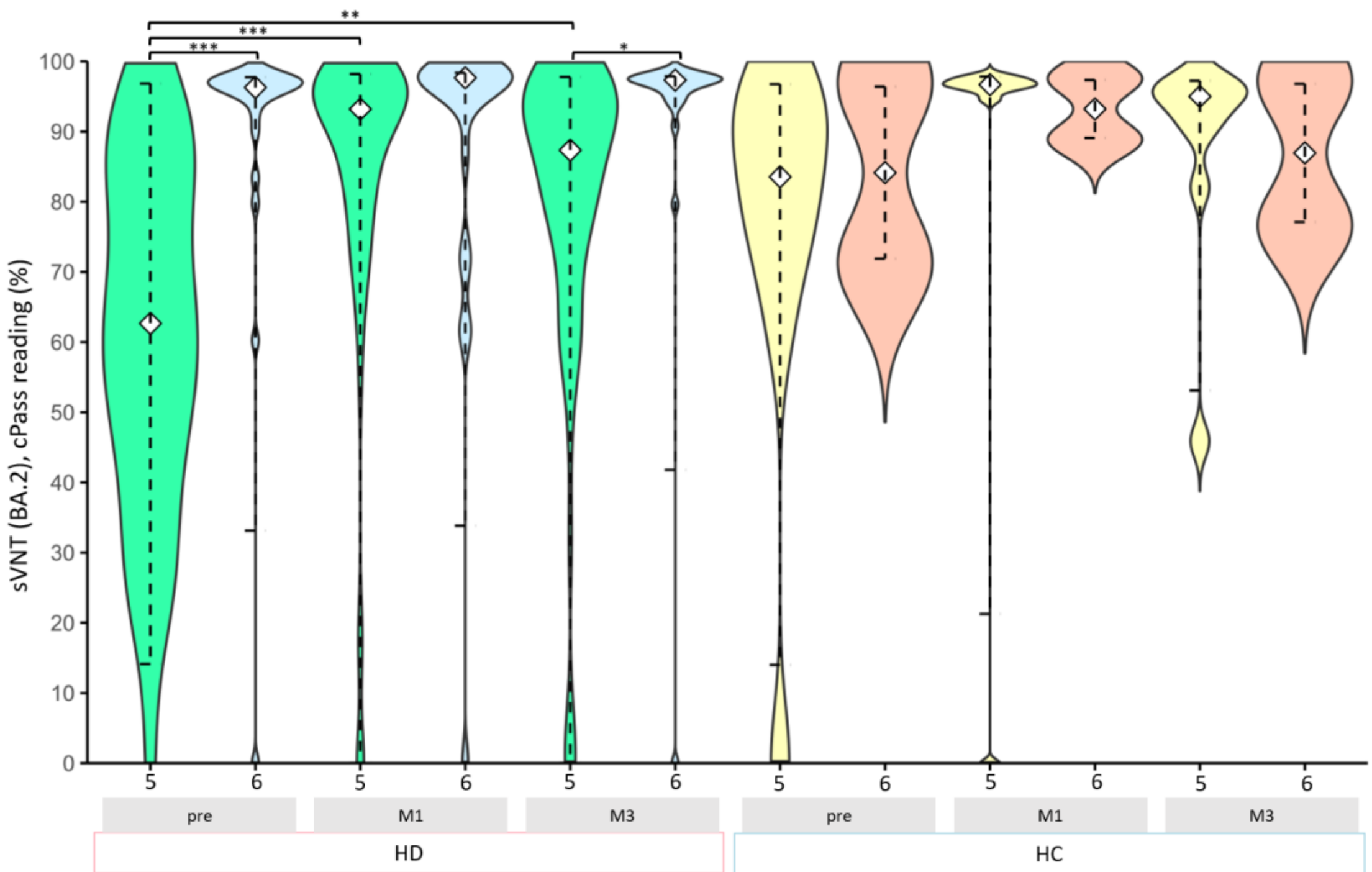
sVNT (BA.2), cPass reading (%)
100
90
80
70
60
50
40
30
20
10
0
**
***
***
*
5
6
5
6
5
6
5
6
5
6
5
6
pre
M1
M3
pre
M1
M3
HD
HC

(E)

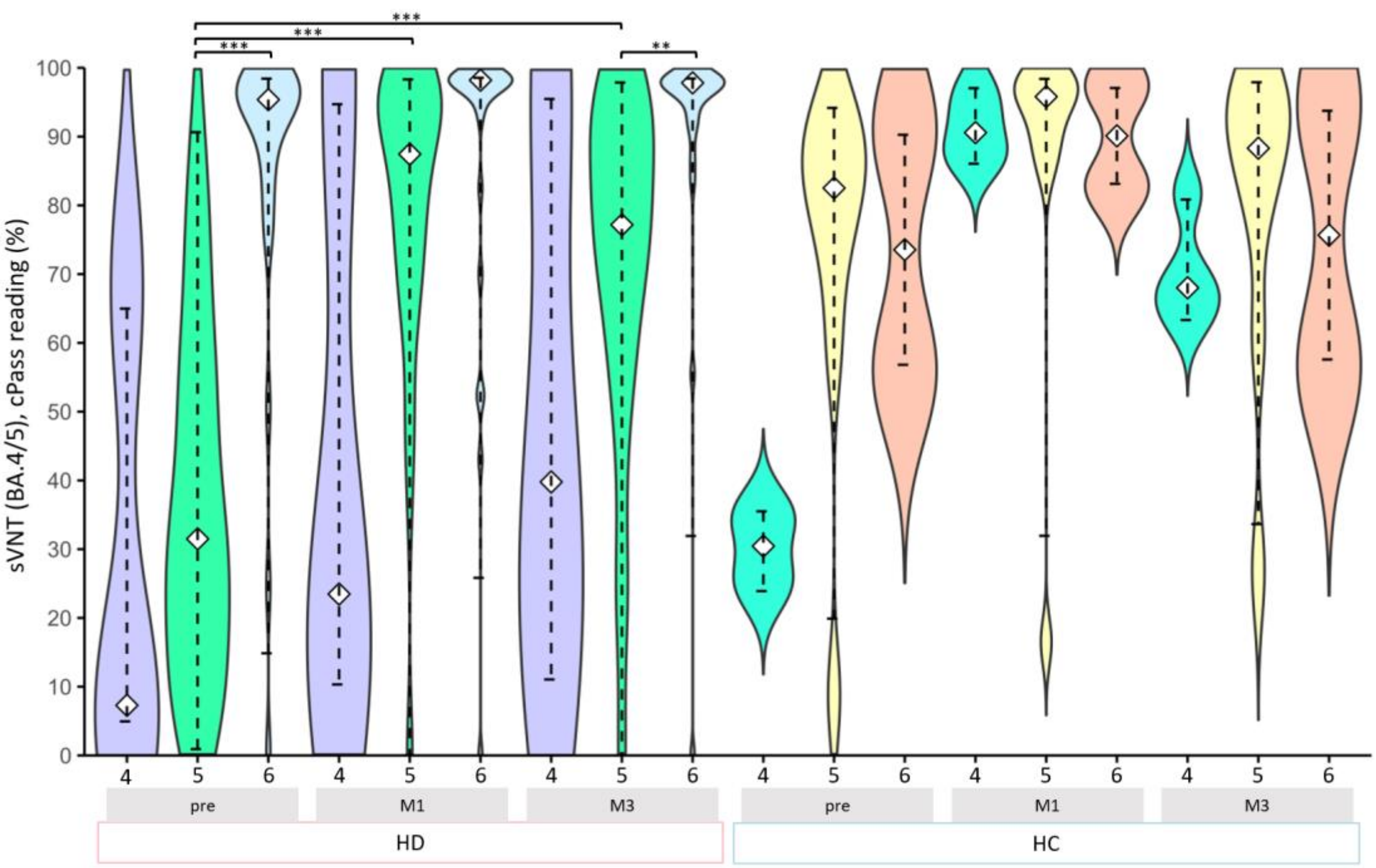

***
***
***
**
sVNT (BA.4/5), cPass reading (%)
100
90
80
70
60
50
40
30
20
10
0
4
5
6
4
5
6
4
5
6
4
5
6
4
5
6
4
5
6
pre
M1
M3
pre
M1
M3
HD
HC
